# Lysophosphatidic Acid Mediates Imiquimod-Induced Psoriasis-like Symptoms by Promoting Keratinocyte Proliferation through LPAR1/ROCK2/PI3K/AKT Signaling Pathway

**DOI:** 10.3390/ijms221910777

**Published:** 2021-10-05

**Authors:** Donghee Kim, Hyo-Jin Kim, Jin-Ok Baek, Joo-Young Roh, Hee-Sook Jun

**Affiliations:** 1 Lee Gil Ya Cancer and Diabetes Institute, Gachon University, Incheon 21999, Korea; dh2388@gachon.ac.kr; 2 College of Pharmacy and Gachon Institute of Pharmaceutical Sciences, Gachon University, Incheon 21936, Korea; hyooojin@gc.gachon.ac.kr; 3 Department of Dermatology, Gachon University College of Medicine, Gil Medical Center, Incheon 21565, Korea; jobaek79@gmail.com (J.-O.B.); jyroh1@gilhospital.com (J.-Y.R.); 4 Gachon Medical Research Institute, Gil Hospital, Incheon 21565, Korea

**Keywords:** psoriasis, keratinocyte, proliferation, lysophosphatidic acid (LPA), Ki16425

## Abstract

Psoriasis is a chronic inflammatory skin disease. Recently, lysophosphatidic acid (LPA)/LPAR5 signaling has been reported to be involved in both NLRP3 inflammasome activation in macrophages and keratinocyte activation to produce inflammatory cytokines, contributing to psoriasis pathogenesis. However, the effect and molecular mechanisms of LPA/LPAR signaling in keratinocyte proliferation in psoriasis remain unclear. In this study, we investigated the effects of LPAR1/3 inhibition on imiquimod (IMQ)-induced psoriasis-like mice. Treatment with the LPAR1/3 antagonist, ki16425, alleviated skin symptoms in IMQ-induced psoriasis-like mouse models and decreased keratinocyte proliferation in the lesion. It also decreased LPA-induced cell proliferation and cell cycle progression via increased cyclin A2, cyclin D1, cyclin-dependent kinase (CDK)2, and CDK4 expression and decreased p27^Kip1^ expression in HaCaT cells. LPAR1 knockdown in HaCaT cells reduced LPA-induced proliferation, suppressed cyclin A2 and CDK2 expression, and restored p27^Kip1^ expression. LPA increased Rho-associated protein kinase 2 (ROCK2) expression and PI3K/AKT activation; moreover, the pharmacological inhibition of ROCK2 and PI3K/AKT signaling suppressed LPA-induced cell cycle progression. In conclusion, we demonstrated that LPAR1/3 antagonist alleviates IMQ-induced psoriasis-like symptoms in mice, and in particular, LPAR1 signaling is involved in cell cycle progression via ROCK2/PI3K/AKT pathways in keratinocytes.

## 1. Introduction

Psoriasis is a chronic inflammatory skin disorder induced by immune cell infiltrates and epidermal keratinocyte dysregulation in the skin [1,2]. Histologically, psoriatic lesions are characterized by immune cell infiltration and epidermal thickening with hyperproliferative keratinocytes [2,3]. While the pathogenic mechanism of psoriasis remains incompletely understood, previous studies have shown that T helper17 (Th17) cells and Th17-associated cytokines such as interleukin (IL)-17A, IL-22, and IL-23 may play a role in the pathogenic mechanism of psoriasis [4,5]. In particular, recently, biological therapies targeting the IL-23/IL-17 axis in psoriasis have shown high therapeutic efficacy, thus, the immune cells are considered as an important factor in psoriasis development [6,7]. However, it has been reported that keratinocytes also play an important role in the development and progression of psoriasis and are involved in recurrence after biological therapy [3,7,8,9]. 

Imiquimod (IMQ), used for treating genital warts caused by human papilloma virus [10], is a potent TLR7/8 agonist and promotes local and acquired immune responses [11]. Topical IMQ application can induce psoriasis-like dermatitis in mice, which is very similar to human psoriasis lesions, not only histologically but also with respect to the development process [11,12,13]. Thus, this experimental model enabled the finding of new therapies for psoriasis. Recent studies have established pathogenicity and molecular targeting mechanisms for psoriasis treatment through an IMQ-induced mouse model [14,15,16,17,18].

Lysophosphatidic acid (LPA) is a small bioactive phospholipid that regulates numerous cellular responses, such as proliferation, survival, and migration, mediated through six G-protein-coupled receptors (GPCRs; LPA receptors 1–6) in various cell types [19,20]. Recently, LPA has been reported to be involved in the manifestation of skin symptoms and skin diseases, such as itch [21], atopic dermatitis [22], scleroderma [23], and allergic skin inflammation [24]. Moreover, LPA levels are elevated in skin lesion and plasma of psoriasis patients and IMQ-induced psoriasis-like disease mice [16,18,25]. In this study, we investigated the effect of ki16425, an LPAR1/3 antagonist, in an IMQ-induced psoriasis-like skin disease mouse model and its mechanisms involved.

## 2. Results

### 2.1. Ki16425 Ameliorates Skin Symptoms and Inflammation in IMQ-Induced Psoriasis-like Mice

**Figure 1:**
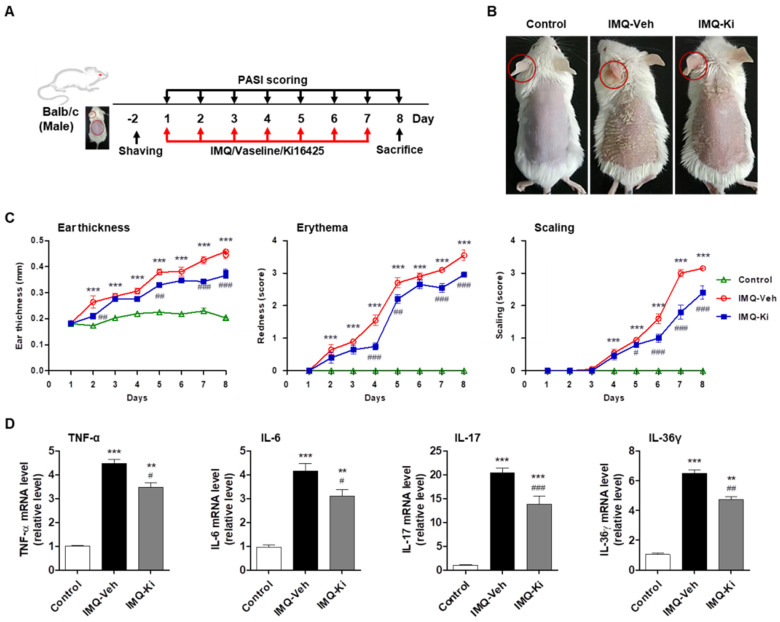
Ki16425 ameliorates skin symptoms and inflammation in imiquimod (IMQ)-induced psoriasis-like mice. (**A**) Experimental design of the IMQ-induced psoriasis-like mouse model and ki16425 treatment. (**B**) IMQ cream or Vaseline was topically applied daily to 7-week-old Balb/c mice for 7 days. Then, representative photographs of back skin were captured, and the phenotypical symptoms of the mouse back skin were observed. The macroscopic appearance is shown. Control, Vaseline-applied normal control; IMQ-Veh, IMQ with vehicle-treated group; IMQ-Ki, IMQ with ki16425-treated group. (**C**) Ear thickness and Psoriasis Area Severity Index (PASI) scores (redness and scaling) were evaluated during the experimental periods (*n* = 9/group). (**D**) The mRNA expression levels of tumor necrosis factor (TNF)-α, interleukin (IL)-6, IL-17, and IL-36γ in skin from experimental mice were analyzed by quantitative real-time RT-PCR (qRT-PCR) (*n* = 4/group). The data are represented as the mean ± standard error of the mean (SEM). ** *p* < 0.01, *** *p* < 0.005 vs. Control; # *p* < 0.05, ## *p* < 0.01, ### *p* < 0.005 vs. IMQ-Veh.

To investigate the effect of LPAR inhibition on psoriasis-like skin disease development, we used the IMQ-induced psoriasis-like Balb/c mouse model (Figure 1A). The control mice (Control) showed normal clear skin, while the IMQ-treated mice (IMQ-Veh) developed psoriasis-like symptoms such as redness, scaling, and skin thickening (Figure 1B). Administration of 15 mg/kg of ki16425, an LPAR1/3 antagonist, for 7 days (IMQ-Ki) significantly improved psoriasis-like symptoms (Figure 1B). The severity of the psoriasis-like skin lesion was scored using the erythema and scaling parts of the Psoriasis Area Severity Index (PASI), and left ear thickness was measured using calipers. Compared with those in the control group, the IMQ-Veh group increased ear thickness, erythema, and scaling, while ki16425 treatment significantly decreased the score compared with the IMQ-Veh group (Figure 1C). Consistent with these results, the expression of inflammatory cytokines, such as TNF-α, IL-6, IL-17, and IL-36γ, in IMQ-induced psoriasis-like skin lesions was significantly higher than that in the control; however, ki16425 treatment significantly reduced the expression of these genes when compared with that in IMQ-Veh group (Figure 1D).

### 2.2. Ki16425 Decreases Epidermal Thickening and Keratinocyte Proliferation in IMQ-Induced Psoriasis-like Mice

**Figure 2:**
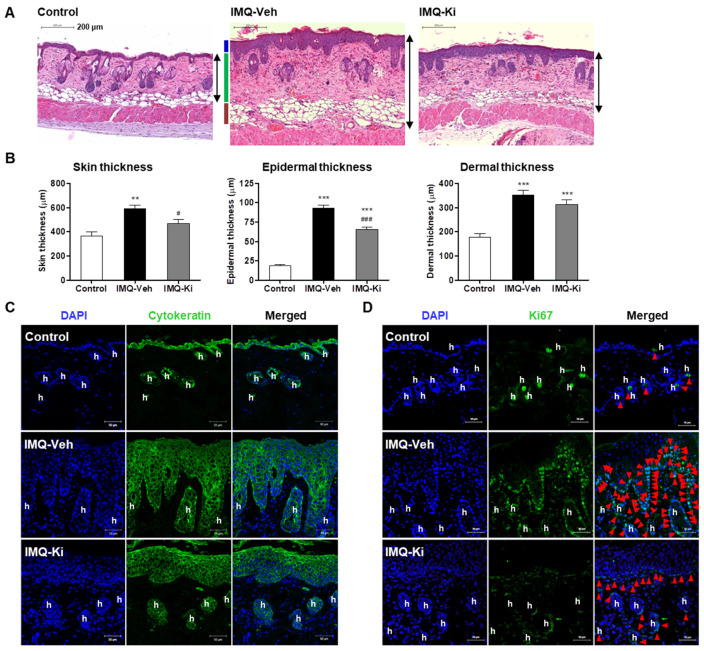
Ki16425 decreases epidermal thickening and keratinocyte proliferation in imiquimod (IMQ)-induced psoriasis-like mice. (**A**) The dorsal skin tissue sections were stained with hematoxylin and eosin (H&E). The representative photographs are shown (original magnification, 200×). (**B**) H&E-stained tissue sections were analyzed for the thickness in skin, epidermis, and dermis. The data are represented as the mean ± standard error of the mean (SEM). ** *p* < 0.01, *** *p* < 0.005 vs. Control; # *p* < 0.05, ### *p* < 0.005 vs. IMQ-Veh. (**C**) The dorsal skin tissue sections were stained for cytokeratin (green) antibody for epithelium and DAPI (blue) for nuclei staining. (**D**) The dorsal skin tissue sections were stained for Ki67 (green) antibody for proliferating cells and DAPI. Red arrowheads indicate green-positive cells. Scale bars: 50 μm. h, hair follicle.

To compare the histology of back skin tissue, we performed hematoxylin and eosin (H&E) staining and measured its thickness. The thickness of the whole skin and epidermis was significantly higher in the IMQ-Veh group than those in the control group (Figure 2A,B); however, the increase in the thickness of these regions was significantly reduced by ki16425 administration (Figure 2A,B). Although the dermal thickness was also significantly higher in the IMQ-Veh and IMQ-Ki groups than that in the control group, this increase was not reduced by ki16425 (Figure 2A,B). The changes in epidermal thickening were confirmed by epithelium staining using the cytokeratin antibody (Figure 2C). The Ki67-positive cells, which indicate proliferating cells [26], were remarkably increased at the dermal junction of the IMQ-Veh group; however, these cells were significantly decreased in the IMQ-Ki group (Figure 2D). Some cytokeratin-positive or Ki67-positive cells were also observed in hair follicles in the dermal layer (Figure 2C,D). These results suggest that LPAR1/3 inhibition alleviates IMQ-induced psoriasis-like symptoms through inhibiting keratinocyte proliferation.

### 2.3. Ki16425 Decreases LPA-Induced HaCaT Cell Proliferation

**Figure 3:**
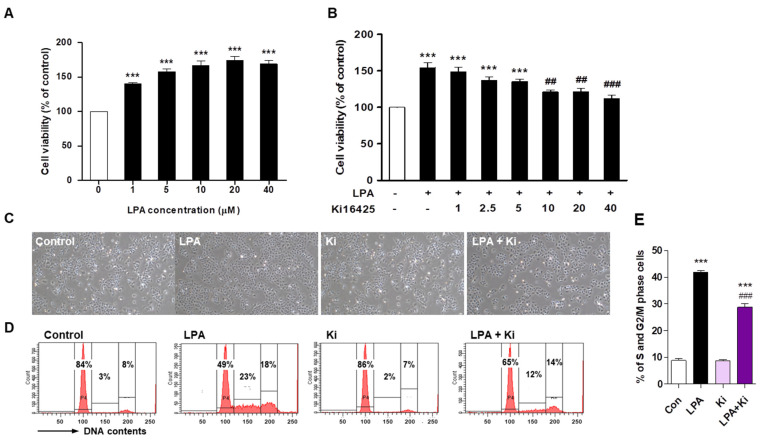
Ki16425 decreases lysophosphatidic acid (LPA)-induced HaCaT cell proliferation. HaCaT cells were seeded and starved in serum-free media containing 0.1% fatty-acid-free bovine serum albumin (FAF-BSA) for 12 to 16 h. (**A**) The cells were treated with 1, 5, 10, 20, and 40 μM LPA for 24 h, and cell viability was analyzed by CCK8 assay. (**B**) The cells were treated with or without 10 μM LPA and indicated ki16425 concentration for 24 h, and cell viability was examined by CCK8 assay. The results are presented as a percentage of control. (**C**–**E**) The cells were treated with 10 μM LPA in the presence or absence of 10 μM ki16425 for 24 h. (**C**) The cell morphology was observed using light microscopy. Original magnification, 100×. (**D**) Cell cycle phase distribution was analyzed using flow cytometry after staining with propidium iodide for measurement of the cellular DNA content. The values in the representative flow cytometry histograms indicate the percentages of cells in G0/G1, S, and G2/M phases of the cell cycle, in that order. (**E**) The percentage of cells in the S and G2/M phase was calculated as the sum of each phase from (**D**). The data are represented as the mean ± standard error of the mean (SEM) of results obtained from three independent experiments. *** *p* < 0.005 vs. Control; ## *p* < 0.01, ### *p* < 0.005 vs. LPA.

To investigate whether LPA affects HaCaT cell proliferation, we evaluated cell growth after LPA treatment. LPA significantly increased the HaCaT cell proliferation concentration-dependently (Figure 3A). We found that 10 μM LPA increased cell proliferation by 165% compared to that in the control. Ki16425 significantly decreased this LPA-induced HaCaT cell proliferation in a concentration-dependent (from 10 μM) manner (Figure 3B). The HaCaT cell morphology also showed the LPA-induced proliferation and inhibition by ki16425 of the LPA-induced proliferation (Figure 3C). The cell cycle analysis showed that the cell portion of S and G2/M phase was significantly increased by LPA, which was significantly decreased by ki16425 (Figure 3D,E). These results suggest that LPA induces keratinocyte proliferation and LPAR1/3 blockage inhibits LPA-induced cell proliferation.

### 2.4. Ki16425 Decreases LPA-Induced Expression of Cyclin A2 and D1, Cyclin Dependent Kinase (CDK)2, and CDK4 in HaCaT Cells

**Figure 4:**
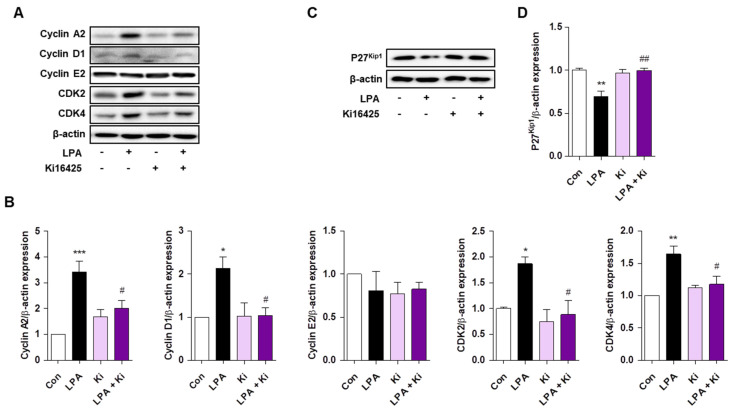
Ki16425 decreases lysophosphatidic acid (LPA)-induced expression of cyclin A2 and D1, CDK2, and CDK4 in HaCaT cells. HaCaT cells were seeded in 60-mm dishes and starved with serum-free media containing 0.1% fatty-acid-free bovine serum albumin (FAF-BSA) for 12 to 16 h. The cells were treated with 10 μM LPA in the presence or absence of 10 μM ki16425 for 6 h. Cyclin A2, D1, and E2, CDK2, CDK4, and p27^Kip1^ protein levels were analyzed by Western blotting, quantified using ImageJ software, and normalized to those of β-actin. Representative images of the blots (**A**,**C**) and relative densitometric bar graphs (**B**,**D**) are shown. The data are presented as the mean ± standard error of the mean (SEM) of results obtained from three to four independent experiments. * *p* < 0.05, ** *p* < 0.01, *** *p* < 0.005 vs. Con; # *p* < 0.05, ## *p* < 0.01 vs. LPA.

LPA induces cell cycle progression and regulates the expression of cell cycle progression related factors such as cyclins and CDKs [27,28]. As we found that LPA treatment increased the HaCaT cell proliferation, we investigated the expression of cell cycle progression related factors. Cyclin A2 and D1 expression was significantly increased by LPA in HaCaT cells, but cyclin E2 expression was not changed (Figure 4A,B). Ki16425 significantly decreased the LPA-induced expression of cyclin A2 and D1 (Figure 4A,B). In addition, CDK2 and CDK4 expression was significantly increased by LPA, which was significantly inhibited by the ki16425 co-treatment (Figure 4A,B). Consistent with the expression pattern of cyclins and CDKs, p27^Kip1^ expression was reduced by LPA and restored by ki16425 co-treatment in HaCaT cells (Figure 4C,D). These results suggest that LPA induces keratinocyte proliferation through regulating the expression of cell cycle progression control factors.

### 2.5. LPAR1 Mediates LPA-Induced HaCaT Cell Proliferation

As ki16425 is an antagonist of LPAR1 and LPAR3, and with moderate activity against LPAR2 [29], we investigated which of these LPARs mediates keratinocyte proliferation.

**Figure 5:**
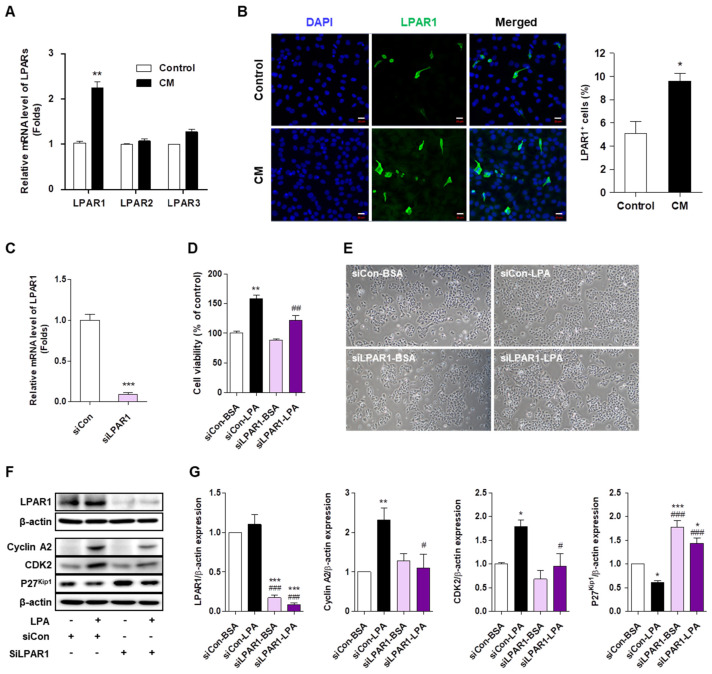
LPAR1 mediates the lysophosphatidic acid (LPA)-induced HaCaT cell proliferation. (**A**) HaCaT cells were seeded in six-well plates and treated with cytokine mixture (CM; 20 ng/mL tumor necrosis factor (TNF)-α and 10 ng/mL interleukin (IL)-6) for 4 h. LPAR1, LPAR2, and LPAR3 mRNA expression was analyzed by qRT-PCR. (**B**) HaCaT cells were seeded in four-well chambers and treated with CM for 24 h. Immunocytochemical analysis was performed using an anti-LPAR1 antibody and an Alexa Fluor 488-conjugated secondary antibody. The nuclei were counterstained with DAPI (blue). Representative staining images (left panel) and percentage of LPAR1 positive cells were quantified using confocal microscope (original magnification, 400×; the scale bars represent 20 μm). (**C**–**E**) HaCaT cells were transfected with a control siRNA (siCon) or LPAR1 siRNA (siLPAR1) for 6 h and treated with LPA for 24 h. (**C**) The mRNA expression of LPAR1 was analyzed by qRT-PCR. (**D**) Cell viability was analyzed by CCK8 assay and calculated as a percentage relative to that of siCon-BSA cells. (**E**) The cell morphology was observed by light microscopy. Original magnification, 100×. (**F**,**G**) HaCaT cells were transfected with siCon or siLPAR1 for 6 h and treated with LPA for 6 h. The levels of the proteins LPAR1, cyclin A2, CDK2, and p27^Kip1^ were analyzed by Western blotting, quantified using ImageJ software, and normalized to those of β-actin. (F) Representative images of the blots and (**G**) relative densitometric bar graphs are shown. The data are represented as the mean ± standard error of the mean (SEM) of results obtained from three to four independent experiments. * *p* < 0.05, ** *p* < 0.01, *** *p* < 0.005 vs. Control or siCon or siCon-BSA; # *p* < 0.05, ## *p* < 0.01, ### *p* < 0.005 vs. siCon-LPA.

To mimic the IMQ-induced inflammatory condition, HaCaT cells were cultured in the presence of inflammatory cytokine mixture (CM; TNF-α and IL-6) for 4 h, and LPAR1–3 mRNA expression was examined. Only LPAR1 mRNA expression in CM-treated cells was higher than that in the control cells (Figure 5A). LPAR2 and LPAR3 expression was not changed by CM treatment. We also confirmed using immunohistochemical staining that LPAR1 expression in CM-treated cells was significantly higher than that in the control cells (Figure 5B). These results suggest that LPAR1 expression was induced in response to inflammatory cytokines in keratinocytes. 

To confirm that LPAR1 regulates LPA-induced cell proliferation, HaCaT cells were transfected with LPAR1 siRNA and then treated with LPA. Compared to control siRNA-transfected cells, LPAR1 expression was significantly reduced in LPAR1 siRNA-transfected cells (Figure 5C). LPA-induced proliferation was significantly decreased by LPAR1 knockdown from that in control siRNA-transfected cells (Figure 5D,E). Consistent with this, LPAR1 knockdown also significantly decreased the expression of cyclin A2 and CDK2 and significantly increased p27^Kip1^ expression compared to that in LPA-treated control siRNA-transfected cells (Figure 5F,G). These results indicate that LPA/LPAR1 signaling regulates the LPA-induced HaCaT cell proliferation.

### 2.6. LPA Promotes Cell Cycle Progression through ROCK2 and PI3K/AKT Signaling in HaCaT Cells

**Figure 6:**
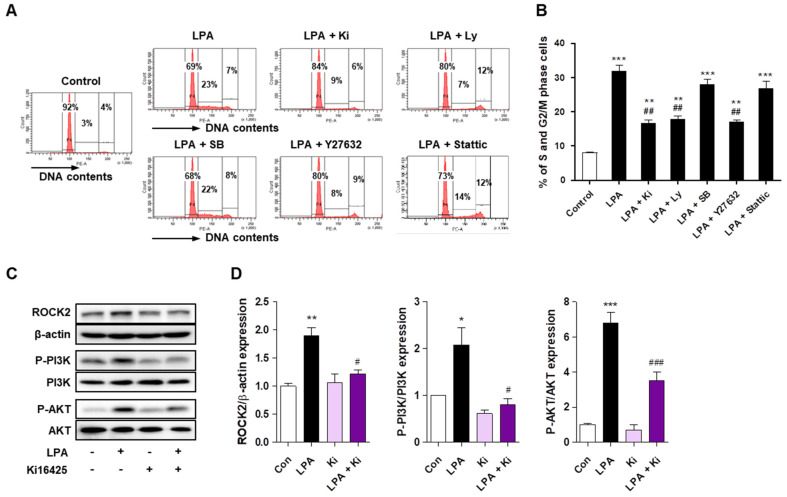
Lysophosphatidic acid (LPA) promotes the cell cycle progression through ROCK2 and PI3K/AKT signaling in HaCaT cells. (**A**,**B**) HaCaT cells were pretreated with Ly294002 (Ly; 10 μM), SB203580 (SB; 10 μM), Y27632 (1 μM), or Stattic (10 μM) for 1 h, and subsequently treated with 10 μM LPA for 24 h, or cells were treated with 10 μM LPA and 10 μM ki16425 simultaneously for 24 h. Cell cycle phase distribution was analyzed using flow cytometry after staining with propidium iodide for measurement of the cellular DNA content. (**A**) The values in the representative flow cytometry histograms indicate the percentages of cells in G0/G1, S, and G2/M phases of the cell cycle, in that order. (**B**) The percentage of cells in the S and G2/M phase was calculated as the sum of each phase from (**A**). (**C**,**D**) The cells were treated with 10 μM LPA in the presence or absence of 10 μM ki16425 for 30 min (for ROCK2 and p-PI3K) and 60 min (for p-AKT). The protein levels of ROCK2, p-PI3K, and p-AKT were analyzed by Western blotting, quantified using ImageJ software, and normalized to those of β-actin, total PI3K, and total AKT, respectively. (**C**) Representative images of the blots and (**D**) relative densitometric bar graphs are shown. The data are presented as the mean ± standard error of the mean (SEM) of results obtained from three independent experiments. * *p* < 0.05, ** *p* < 0.01, *** *p* < 0.005 vs. Con; # *p* < 0.05, ## *p* < 0.01, ### *p* < 0.005 vs. LPA.

Previous studies have reported that LPA induces cell proliferation through various intracellular signaling pathways such as ROCK [27,30], Stat3 [27], PI3K [30], and mitogen-activated protein kinase (MAPK) [31] in various cell types. Therefore, to determine the intracellular signaling pathway involved in LPA-induced HaCaT cell proliferation, we investigated the effect of the inhibitors of these signaling molecules in the LPA-induced cell cycle changes. Similar to the ki16425 treatment in HaCaT cells, pretreatment with PI3K inhibitor (Ly294002) and ROCK inhibitor (Y27632), but not p38 MAPK inhibitor (SB203580) and stat3 inhibitor (Stattic), decreased the LPA-induced increase in S and G2/M phase cells (Figure 6A,B). Based on the results of the inhibitor treatment, we investigated whether ROCK2 and PI3K/AKT were activated by the LPA treatment in HaCaT cells. LPA significantly increased the expression of ROCK2, phospho-PI3K, and phospho-AKT compared to that in the control cells (Figure 6C,D). However, ki16425 significantly suppressed the expression of these intracellular signaling factors compared to that in the LPA-treated cells (Figure 6C,D). These results suggest that LPA promotes the cell cycle progression via ROCK2 and PI3K/AKT signaling pathways in HaCaT cells.

### 2.7. Ki16425 Decreases the Epidermal Expression of ROCK2 and p-AKT in IMQ-Induced Psoriasis-like Mice 

**Figure 7:**
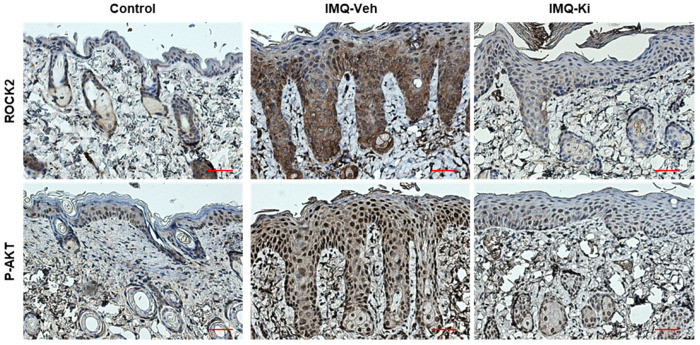
Ki16425 decreases the epidermal expression of ROCK2 and p-AKT in IMQ-induced psoriasis-like mice. Immunohistochemical detection of ROCK2 and p-AKT was performed in the skin tissues using the specific antibodies and DAB staining kit. The nuclei were counterstained with hematoxylin. The representative images are depicted (original magnification, 200×, *n* = 3/group). Scale bars: 50 μm.

To confirm the effect of the ki16425 treatment on the epidermal expression of ROCK2 and p-AKT in IMQ-induced psoriasis-like mice, we performed immunohistochemical staining to analyze the expression of these molecules in skin tissues. Consistent with the results in HaCaT cells, the epidermal expression of ROCK2 and p-AKT was significantly increased in IMQ-induced psoriasis-like mice compared to that in the control mice; however, the increase in the expression of these molecules was significantly inhibited by the ki16425 treatment (Figure 7). These results confirm that the LPA-induced expression of ROCK2 and p-AKT is involved in the keratinocyte proliferation of psoriasis-like skin disease not only in vitro but also in vivo. 

## 3. Discussion

Psoriasis is a chronic inflammatory skin disease characterized by keratinocyte proliferation in epidermis and immune cell infiltration [1,32]. Over the past 20 years, immune cells have been considered as the main cause of psoriasis due to the high therapeutic efficacy of biological agents such as neutralizing antibodies targeting the IL-23/IL-17 axis [6,7]. However, keratinocytes not only trigger psoriasis onset but also act as executors in cytokine-mediated multimolecular networks [7]. Various cytokines secreted by the immune cells in the lesion can contribute to keratinocyte hyperproliferation by stimulating keratinocytes, and hyperproliferative keratinocytes subsequently produce massive proinflammatory cytokines to maintain or further amplify the inflammatory response [3,7,8]. In addition, keratinocytes regulate psoriasis relapse [7,9]. As these suggest that keratinocytes may be an important target in psoriasis treatment, the strategies to inhibit keratinocyte hyperproliferation are considered potentially useful for psoriasis treatment.

LPA promotes skin barrier function by intrinsically regulating keratinocyte differentiation through LPAR1/LPAR5 signaling [33]. In skin diseases, however, LPA acts as a pruritogenic mediator through LPAR1 in atopic dermatitis model [24] and induces itch through LPAR5 [21]. It has recently been reported that the plasma LPA levels in psoriasis patients are significantly higher than that in the control [18], and the LPA levels in skin lesions and plasma of IMQ-induced psoriasis mice [16,18] are also significantly higher than that in the control. These suggest that LPA/LPAR signaling may play an important role in psoriasis progression. Moreover, LPAR5 is involved in NLRP3 inflammasome activation in macrophages of psoriatic lesions [16] and also keratinocyte activation to produce inflammatory cytokines [18], contributing to psoriasis pathogenesis. When we tested the effect of ki16425, an LPAR1/3 antagonist, on IMQ-induced psoriasis-like mice, we found that it ameliorated IMQ-induced psoriasis-like symptoms, mainly by inhibiting keratinocyte hyperproliferation (Figure 1 and Figure 2). We found that ki16425 significantly, but not completely, inhibited mRNA expression of inflammatory cytokines, such as TNF-α, IL-6, IL-17, and IL-36γ, in skin lesions, as well as PASI levels (Figure 1). IL-36γ, an IL-1 family cytokine, is secreted from keratinocytes activated by TNF-α and IL-17 in the psoriatic epidermis to induce T helper subset polarization and promote self-amplifying loops [34,35]. Therefore, it has been considered that the decreased IL-36γ expression in the lesion by ki16425 treatment reflects that inhibition of LPAR1/3 signaling suppresses the activity of keratinocytes as well as immune cells. 

Histological changes in skin lesions showed that the Ki67-positive cells representing epidermal keratinocyte proliferation are mainly found at the dermal junction in IMQ-induced psoriatic skin lesions as reported previously [36,37]. Moreover, the ki16425 treatment significantly reduced epidermal thickening due to IMQ-induced keratinocyte hyperproliferation (Figure 2), suggesting that LPAR1/3 inhibition might decrease keratinocyte proliferation. Indeed, our in vitro results showed that the LPA-induced HaCaT cell proliferation was inhibited by ki16245 treatment (Figure 3). S and G2/M phase cell populations were significantly increased in LPA-treated HaCaT cells, which were reduced by ki16425 treatment, suggesting that the cell cycle is promoted by LPA.

The cell cycle phase transitions are regulated by the balance between the activities or levels of cyclins, CDKs, and CDK inhibitors [38]. The expression of cyclin A2, which is responsible for S phase and G2/M transition, was significantly increased in LPA-treated HaCaT cells (Figure 4), as reported in an LPA-treated colorectal cancer cell line [27]. The expression of cyclin D1, which is an essential regulator of the G1 to S phase transition, was also significantly increased in LPA-treated HaCaT cells, as reported in an LPA-treated renal mesangial cell line [39]. During cell cycle progression, cyclin A2 and cyclin D1 act in combination with CDK2 and CDK4, respectively [38]. Consistent with cyclin A2 and D1 expression, LPA significantly increased CDK2 and CDK4 expression in HaCaT cells, and ki16425 treatment significantly decreased these LPA-induced increases (Figure 4). Similarly, cyclin D1, CDK2, and CDK4 expression is increased in psoriatic lesions of psoriasis patients [40]. In addition to cell cycle regulation, cyclin A2 plays a role in cytoskeletal rearrangement and cell migration [41,42]. Since excess actin cytoskeleton organization is found in psoriatic skin lesions [43], cyclin A2 might also be involved in the abnormal actin cytoskeletal organization in the keratinocytes during psoriasis. Consistently, the expression of p27^Kip1^, a negative CDK2 regulator [44], decreased in HaCaT cells by LPA treatment and was restored by ki16425 treatment (Figure 4). In agreement with this result, p27^Kip1^ expression reduces in psoriatic epidermis of psoriasis patients [45]. 

Although ki16425 is an antagonist of LPAR1 and LPAR3 and has moderate activity against LPAR2 [26], we found that only LPAR1 expression increased under inflammatory conditions in HaCaT cells (Figure 5). Therefore, to verify the involvement of LPAR1 signaling in LPA-induced keratinocyte proliferation, we used RNA interference system using LPAR1 siRNA. LPAR1 knockdown in HaCaT cells significantly inhibited the LPA-induced expression of cell cycle control factors and significantly, but not completely, suppressed proliferation (Figure 5). This suggests that LPA-induced HaCaT cell proliferation is partially promoted through LPAR1. Since LPAR5 is expressed in keratinocytes [18], it is possible that LPAR5 might also be involved in keratinocyte proliferation. Further studies are needed to clarify the role of LPAR5 in the keratinocyte proliferation. 

We then investigated the signaling molecules involved in LPA-induced cell cycle progression. Although LPA is known to elicit cellular responses through various signaling pathways [19], ROCK, STAT3, p38 MAPK, and PI3K/AKT pathways are specifically involved in LPA-induced proliferation in various cell types, such as corneal endothelial cells, colorectal cancer cells, and renal mesangial cells [27,30,39]. Among them, ROCK2 and PI3K/AKT signaling molecules were involved in LPA-induced HaCaT cell proliferation (Figure 6). Consistent with the results in HaCaT cells, the expression of ROCK2 and p-AKT was significantly increased in the skin of IMQ-induced psoriasis-like mice; however, the increase in the expression of these molecules was decreased by ki16425 treatment. Similar to our findings, it has been recently reported that human keratinocytes promoted proliferation through the PI3K/AKT signaling pathway under EGFR-stimulated conditions [46], and epidermal *ROCK2* overexpression in mice induces AKT1 activation and epidermal cell proliferation [47]. 

**Figure 8:**
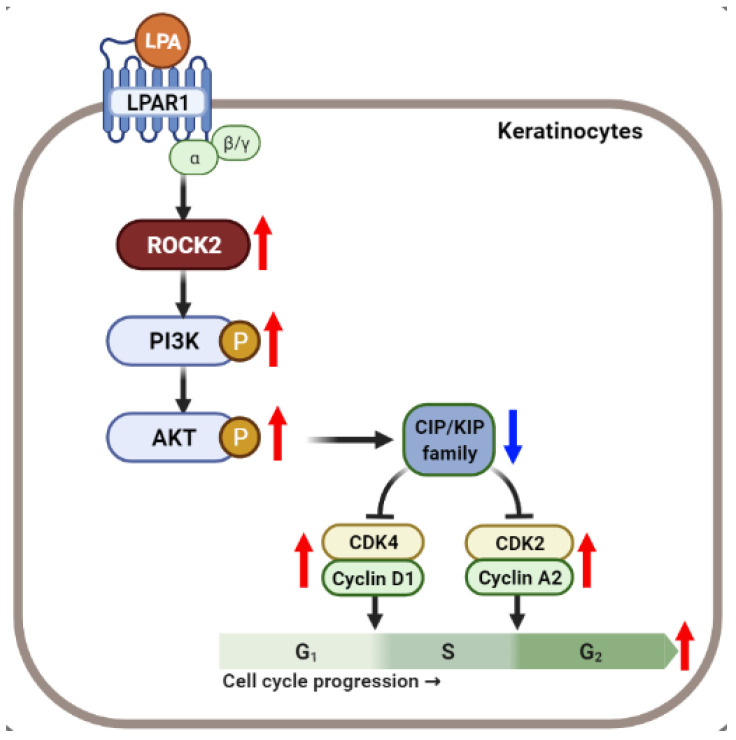
Schematic representation of the mechanism of LPA-mediated cell cycle progression via ROCK2/PI3K/AKT signaling in keratinocytes. Created with BioRender.com. Red and blue arrows indicate upregulated and downregulated responses, respectively.

In summary, our data demonstrate that LPA induced ROCK2 and PI3K/AKT signaling pathway-mediated cell cycle progression and proliferation in keratinocytes (Figure 8). Ki16425 or LPAR1 knockdown inhibited these processes and prevented cell proliferation, thus contributing to the attenuation of IMQ-induced psoriasis-like symptoms. Taken together, we suggest that LPA-induced keratinocyte proliferation could be one of the mechanisms underlying psoriasis development and combination therapy targeting LPAR1 and LPAR5 might be one of the options for psoriasis treatment.

## 4. Materials and Methods

### 4.1. Chemicals and Reagents

Ki16425 and DMSO were purchased from Biobyt Ltd. (Cambridge, UK) and Sigma-Aldrich Co. (St. Louis, MO, USA), respectively. Aldara cream (5% imiquimod, Aldara^®^; 3M Pharmaceuticals, Loughborough, UK) was kindly supplied by Dong-Ah Pharmaceutical Co. (Seoul, Korea). LPA was purchased from Avanti Polar Lipids Inc. (Alabaster, AL, USA). We used Ly294002 (10 μM, Sigma-Aldrich Co.), SB203580 (10 μM; Enzo Life Sciences, Farmingdale, NY, USA), Y27632 (1 μM, American Type Culture Collection, Manassas, VA, USA), and Stattic (10 µM, Sigma-Aldrich Co.) to inhibit phosphoinositide 3-kinase (PI3K), p38 MAPK, Rho-associated, coiled-coil containing protein kinase (ROCK), and signal transducer and activator of transcription 3 (STAT3), respectively.

### 4.2. Animals and Experimental Psoriasis Induction

Six-week-old male Balb/c mice were purchased from Korea Research Institute of Bioscience and Biotechnology (KRIBB, Daejeon, South Korea) and adapted for one week before starting the experiments. The mice were maintained at the animal facility of Lee Gil Ya Cancer and Diabetes Institute, Gachon University (Incheon, Korea). All animal experiments were carried out according to a protocol approved by the Institutional Animal Care and Use Committee at Lee Gil Ya Cancer and Diabetes Institute, Gachon University (LCDI-2017-0100). 

The mice were grouped as normal control group (Control), IMQ with vehicle-treated group (IMQ-Veh), and IMQ with ki16425-treated group (IMQ-Ki). The mice in IMQ-Veh and IMQ-Ki groups applied 83 mg of 5% IMQ cream (3M Pharmaceuticals) daily on their shaved dorsal skins and left ear folds. The mice in the Control group applied a similar daily dose of vehicle cream (Vaseline cream) [48]. After IMQ application, the mice were daily injected with 15 mg/kg of ki16425 or an equivalent volume of DMSO intraperitoneally for seven consecutive days. The control mice as a negative group were treated with only Vaseline cream.

### 4.3. Analysis of Dermatitis Severity

All animals were daily assessed for the severity of the psoriasis-like skin condition, using two elements of the PASI, to assign a score of 0–4 (0, none; 1, mild; 2, moderate; 3, severe; 4, very severe) for erythema and scaling [48]. The thickness of the left ear fold was measured using a caliper (accuracy ± 0.02 mm; Mitsutomo Ltd., Nagoya, Japan). Based on H&E staining, the thickness of the skin, epidermis, and dermis of the dorsal skin was measured by microscopic image analysis.

### 4.4. Quantitative Real-Time RT-PCR (qRT-PCR) Analysis

Total RNA was extracted from skin tissues or cells using RNAiso Plus reagent (Takara Bio Inc., Kyoto, Japan), according to the manufacturer’s instructions. The cDNA was synthesized using a PrimeScript First Strand cDNA Synthesis Kit (Takara Bio Inc.) and qRT-PCR was performed using SYBR Premix Ex Taq II (Takara Bio Inc.) on a CFX384^TM^ Real-Time PCR System (Bio-Rad, Hercules, CA, USA). The relative gene expression levels were normalized to that of cyclophilin and calculated using the 2^−ΔΔCT^ relative quantification method. The sequences of the primer pairs used for PCR are enlisted in Supplementary Table S1.

### 4.5. Histological Analysis

The dorsal skin samples were removed from mice, fixed in 10% neutral-buffered formalin (Sigma-Aldrich Co.), and embedded in a paraffin block. Tissue sections (4 μm) were deparaffinized in xylene and rehydrated in a graded alcohol solution series. The sections were stained with hematoxylin (Sigma-Aldrich Co.) and eosin (Sigma-Aldrich Co.) (H&E) and observed using a light microscope (Olympus, AX70, TR-62A02; Olympus Co., Tokyo, Japan).

For immunohistochemical staining, skin sections were subjected to heat-induced antigen retrieval using citrate buffer (10 mM citric acid, 0.05% Tween 20, pH 6.0) and blocked in Protein Block Serum-Free Ready-To-Use solution (Dako North America, Inc.; Carpinteria, CA, USA) at 25 °C for 1 h. Then, the sections were incubated overnight at 4 °C with cytokeratin antibody (Z0622; Dako North America, Inc.) or Ki67 antibody (ab15580; Abcam, Cambridge, MA, USA) diluted to 1:200 using Antibody Diluent (Dako North America, Inc.). After washing with PBS, the sections were incubated using anti-rabbit Alexa 488 secondary antibody (Abcam) diluted to 1:200 using Antibody Diluent. The nuclei were counterstained with 4′,6′-diamidino-2-phenylindole (DAPI) (Invitrogen, San Diego, CA, USA) diluted to 1:1000 using PBS at 25 °C for 5 min and mounted with a Fluorescence Mounting Medium (Dako North America, Inc.). These sections were observed using a confocal microscope (Carl Zeiss Inc., Oberkochen, Germany).

For analyzing the expression of ROCK2 and phosphorylated-AKT, immunohistochemical staining was performed using Polink-2 Plus HRP Rabbit with DAB Kit (D39-18, GBI Labs. Inc., Bothell, DC, USA) or Polink-2 Plus HRP Mouse with DAB Kit (D37-18, GBI Labs. Inc.), respectively, according to the manufacturer’s protocol. The primary antibodies used were anti-ROCK2 (sc-5561; Santa Cruz Biotech., Santa Cruz, CA, USA) and anti-phosphorylated-AKT (sc-514032; Santa Cruz Biotech.). The nuclei were counterstained with hematoxylin (Sigma-Aldrich Co.). The sections were subsequently dehydrated, mounted, and observed under a light microscope (Carl Zeiss Inc.).

### 4.6. Cell Culture and Treatment

The HaCaT cell line, a human keratinocyte cell line, was obtained from the CLS Cell Lines Service GmbH (Eppelheim, Germany) and grown in Dulbecco’s modified Eagle medium (DMEM; Welgene, Daegu, Korea) supplemented with 10% fetal bovine serum (FBS), 100 unit/mL penicillin G, and 100 µg/mL streptomycin at 37 °C in 5% CO_2_.

To investigate the effect of LPA, HaCaT cells (3 × 10^5^ cells) were plated on 60 mm dishes and cultured for 24 h. Since serum contains LPA, according to a previous our report on treatment with LPA in mesangial cells [39], the medium was switched to serum-free medium (SFM) containing 0.1% fatty acid-free bovine serum albumin (FAF-BSA, Sigma-Aldrich Co.), and incubated for 16–18 h. The cells were then treated with 10 μM LPA in the presence or absence of 10 μM ki16425. To determine the signaling mechanisms involved, the cells were pretreated with Ly294002, SB203580, Y27632, or Stattic for 1 h, and subsequently treated with 10 μM LPA for 24 h.

### 4.7. Cell Viability Assay

Cell viability was analyzed using a D-Plus™ CCK8 cell viability assay kit (Dongin LS, Seoul, Korea) in accordance with the manufacturer’s protocol. After incubation in SFM, the HaCaT cells were treated with 1, 5, 10, 20, and 40 μM LPA for 24 h. To test the effects of ki16425 on LPA-induced cell proliferation, the cells were treated with or without 10 μM LPA and 1–40 μM ki16425 for 24 h. Then, cell viability was analyzed using CCK8 assay and calculated as a percentage relative to that of control cells.

### 4.8. Cell Cycle Analysis

After incubation with LPA in the presence or absence of various inhibitors, HaCaT cells were collected, washed with ice-cold PBS (pH 7.4), and fixed with 80% (*v*/*v*) cold ethanol including 0.1% Tween 20, and incubated overnight at 4 °C. The cells were then washed twice with PBS, resuspended in PBS containing 10 μg/mL RNase A (Takara Bio Inc.) and propidium iodide (PI, 50 μg/mL, Sigma-Aldrich Co.), and incubated at 37 °C for 30 min in dark. The DNA content and cell cycle distribution were analyzed using FACS LSR II equipped with CellQuest software (Becton Dickinson Biosciences, San Jose, CA, USA).

### 4.9. Immunocytochemistry

HaCaT cells (2 × 10^4^ cells/well) were seeded in a four-well chamber and cultured for 18 h. After the medium was replaced with a cytokine mixed medium containing TNF-α (20 ng/mL; PeproTech, Rocky Hill, NJ, USA) and IL-6 (10 ng/mL; PeproTech), the cells were incubated for 24 h, fixed using 5% neutral-buffered formalin for 10 min, and permeabilized using 0.2% Triton X-100 (Sigma-Aldrich Co.) in PBS for 10 min. The cells were then blocked using Protein Block Serum-Free Ready-To-Use solution (Dako North America, Inc.) at 25 °C for 1 h and incubated with LPAR1 antibody (ab23698; Abcam) diluted at 1:200 in Antibody Diluent (Dako North America, Inc.) at 4 °C overnight. Then, the cells were incubated with anti-Rabbit Alexa 488 secondary antibody (Abcam) diluted at 1:200 in Antibody Diluent. The nuclei were stained with DAPI (Invitrogen) diluted at 1:1000 in PBS, mounted with fluorescence mounting media (Dako North America, Inc.), and observed using a confocal microscope (Carl Zeiss Inc.).

### 4.10. Western Blot Analysis

After incubation with LPA and/or other inhibitors, HaCaT cells were washed with cold PBS (pH 7.4) and lysed using Mammalian Protein Extraction Buffer (GE Healthcare, Milwaukee, WI, USA) supplemented with protease and phosphatase inhibitor cocktail (GenDEPOT, Barker, TX, USA). The protein concentrations were measured using the Pierce BCA protein assay kit (Thermo Fisher Scientific, Rockford, IL, USA). The total proteins (10–50 µg) were separated using sodium dodecyl sulfate polyacrylamide gel electrophoresis (SDS–PAGE) and transferred to polyvinylidene fluoride (PVDF) membranes (Millipore, Billerica, MA, USA), which were incubated with 5% skimmed milk for 1 h at 25°C, and then incubated with primary antibodies overnight at 4 °C. After washing extensively, the membranes were incubated with the horseradish peroxidase-conjugated secondary antibody (Jackson ImmunoResearch, West Grove, PA, USA). The signal was detected using chemiluminescence system LAS-4000 (Fuji Film, Tokyo, Japan) with Immobilon Western Chemiluminescent HRP substrate (Millipore). ImageJ software (NIH, Bethesda, MD, USA) was used to quantify the band intensity of Western blotting. The following primary antibodies were used: anti-p27^Kip1^ (#2552; Cell Signaling Technology (CST); Boston, MA, USA), anti-Cyclin A2 (MA1-154; Thermo Fisher Scientific), anti-Cyclin D1 (sc-450; Santa Cruz Biotech.), anti-Cyclin E2 (sc-28351; Santa Cruz Biotech.), anti-CDK2 (sc-163; Santa Cruz Biotech.), anti-CDK4 (sc-260; Santa Cruz Biotech.), anti-ROCK2 (sc-5561; Santa Cruz Biotech.), p-PI 3-kinase p85α (Tyr508) (sc-12929; Santa Cruz Biotech.), PI 3-kinase p85α (B-9) (sc-1637; Santa Cruz Biotech.), p-Akt1/2/3 (C-11) (sc-514032; Santa Cruz Biotech.), Akt1/2/3 (5C10) (sc-81434; Santa Cruz Biotech.), and anti-β-actin (sc-47778; Santa Cruz Biotech.). β-actin was used as a loading control. Three or four separate experiments were performed with different samples.

### 4.11. Transient Transfection

For small interfering RNA (siRNA) transfection, HaCaT cells were plated and transiently transfected with LPAR1 siRNA (Bioneer Inc., Daejeon, Korea) or scrambled siRNA (Bioneer Inc.) using Lipofectamine RNAiMAX (Invitrogen) according to the manufacturer’s instructions. After 6 h, the medium was replaced with SFM containing 0.1% FAF-BSA and incubated for 16 to 18 h. The cells were then treated with 10 μM LPA for 6 h or left unstimulated.

### 4.12. Statistical Analysis

The statistical analyses were performed by one-way analysis of variance (ANOVA) with Tukey’s multiple comparison test using GraphPad Prism version 7.03 (GraphPad Software Inc., San Diego, CA, USA). The data are presented as the mean ± standard error of the mean (SEM). Statistical significance was considered at *p* < 0.05.

## Data Availability

The data used to support the findings of this study are available from the corresponding author upon request.

## Supplementary Materials

The following are available online at https://www.mdpi.com/article/10.3390/ijms221910777/s1. Table S1: Primer sequences for qRT-PCR PCR.
