# Supplementary material for: Lysophosphatidic Acid Mediates Imiquimod-Induced Psoriasis-like Symptoms by Promoting Keratinocyte Proliferation through LPAR1/ROCK2/PI3K/AKT Signaling Pathway
Source: Int J Mol Sci. 2021 Oct 5;22(19):10777. doi: 10.3390/ijms221910777 (PMC8509620; doi:10.3390/ijms221910777)
Supplement: Supplementary file 1 [file ijms-22-10777-s001.zip › ijms-1381680-supplementary.pdf]

Supplementary Table S1: Primer sequences for qRT-PCR

Gene symbol	Species	Forward primer	Reverse primer
Cyclophilin	Human	TGCCATCGCCAAGGAGTAG	TGCACAGACGGTCACTCAAA
LPAR1	Human	GCAATCGAGAGGCACATTACG	CGGTTGCTCATCCGTGTGT
LPAR2	Human	TGCTACTACAACGAGACCATC	CACCACGACCACATCCTT
LPAR3	Human	GGTCCATAGCAACCTGACCAA	ATGGCCCAGACAAGCAAAAT
Cyclophilin	Mouse	TGGAGAGCACCAAGACAGACA	TGCCGGAGTCGACAATGAT
TNF-α	Mouse	CCAACGGCATGGATCTCAAAGACA	AGATAGCAAATCGGCTGACGGTGT
IL-6	Mouse	TCCAGTTGCCTTCTTGGGACTGAT	AGCCTCCGACTTGTCAAGTGGTAT
IL-17	Mouse	GTGTCAATGCGGAGGGAA	TTCAGGACCAGGATCTCTTGCT
IL-36γ	Mouse	CAGGCCCTTGTGACAGTTCCA	TCCTGTTCAAGAGACTCTGGGT
